# Trigger phosphodiesterases as a novel class of c-di-GMP effector proteins

**DOI:** 10.1098/rstb.2015.0498

**Published:** 2016-11-05

**Authors:** Regine Hengge

**Affiliations:** Institute of Biology/Microbiology, Humboldt-Universität zu Berlin, 10115 Berlin, Germany

**Keywords:** biofilm, cellulose, curli fibres, diguanylate cyclase, EAL domain, second messenger

## Abstract

The bacterial second messenger c-di-GMP controls bacterial biofilm formation, motility, cell cycle progression, development and virulence. It is synthesized by diguanylate cyclases (with GGDEF domains), degraded by specific phosphodiesterases (PDEs, with EAL of HD-GYP domains) and sensed by a wide variety of c-di-GMP-binding effectors that control diverse targets. c-di-GMP-binding effectors can be riboswitches as well as proteins with highly diverse structures and functions. The latter include ‘degenerate’ GGDEF/EAL domain proteins that are enzymatically inactive but still able to bind c-di-GMP. Surprisingly, two enzymatically active ‘trigger PDEs’, the *Escherichia coli* proteins PdeR and PdeL, have recently been added to this list of c-di-GMP-sensing effectors. Mechanistically, trigger PDEs are multifunctional. They directly and specifically interact with a macromolecular target (e.g. with a transcription factor or directly with a promoter region), whose activity they control by their binding and degradation of c-di-GMP—their PDE activity thus represents the c-di-GMP sensor or effector function. In this process, c-di-GMP serves as a regulatory ligand, but in contrast to classical allosteric control, this ligand is also degraded. The resulting kinetics and circuitry of control are ideally suited for trigger PDEs to serve as key components in regulatory switches.

This article is part of the themed issue ‘The new bacteriology’.

## 1. The bacterial nucleotide second messenger c-di-GMP

Over the past decade, bis-(3′,5′)-cyclic di-guanosine-mono-phosphate (c-di-GMP) has emerged as a nearly ubiquitous bacterial nucleotide second messenger [1–5]. In many bacteria, c-di-GMP promotes biofilm formation, i.e. the synthesis of biofilm matrix components such as amyloid fibres or exopolysaccharides and the expression of adhesins and other biofilm-relevant functions. In many species, c-di-GMP also inhibits the expression and/or activity of flagella. However, the notion of c-di-GMP as a signal that inhibits the motile planktonic ‘lifestyle’ and induces the sessile biofilm ‘lifestyle’ is certainly an oversimplification. Thus, planktonic *E. coli* cells grown in liquid culture produce extracellular matrix components when they enter into stationary phase [6,7] and flagella are present and play an important role in *E. coli* biofilms, which contain cells in different physiological states in different zones [8,9]. In addition, c-di-GMP can also regulate virulence gene expression, cell-type differentiation and cell cycle progression in *Caulobacter* and bacterial development, e.g. in *Myxococcus* and *Streptomyces* [10–14].

c-di-GMP is synthesized from GTP by diguanylate cyclases (DGC) characterized by the GGDEF domain (this motif represents the conserved active site or A-site). Most, but not all DGCs also contain a secondary binding site for c-di-GMP (I-site), which allosterically slows down further c-di-GMP synthesis once elevated cellular levels have been reached. Degradation of c-di-GMP is mediated by specific phosphodiesterases (PDEs), which can feature either EAL or HD-GYP domains [15,16]. Many DGCs and PDEs actually feature GGDEF and EAL domains in the same protein, with usually one domain being enzymatically active and the other being degenerate and exerting a regulatory influence. A majority of these enzymes harbour diverse N-terminal sensory input domains that control their activities in response to intra- or extracellular signals. These include two-component receiver, PAS, GAF, globin sensor, various light-sensing as well as distinct membrane-integral MASE, CHASE or GAPES domains [17–21].

c-di-GMP signalling has become a top research priority in the field of molecular microbiology since its function is of unprecedented complexity in bacterial second messenger signalling. In particular, two features have led to novel paradigms, i.e. (i) the multiplicity of DGCs and PDEs in single species [3] and (ii) the diversity of c-di-GMP-sensing effector or receptor components [22,23].

## 2. Multiplicity of DGCs and PDEs and ‘local’ c-di-GMP signalling

Genome sequencing has revealed a surprising abundance of GGDEF/EAL domain-encoding genes in the genomes of many bacterial species, in particular in gamma-proteobacteria [24]. For instance, the pangenome of *E. coli* (as known in 2015) includes 35 genes encoding GGDEF/EAL domains. Among the 29 such genes of the well-characterized laboratory strain *E. coli* K-12, 12 encode DGCs and 13 encode PDEs [21,25]. The multiplicity of DGCs and PDEs in single species as well as frequent observations that knocking out a particular DGC or PDE can lead to a clearcut phenotype—not observed with knock-out mutations in other such genes in the same species—has spurred hypotheses about ‘local signalling’ based on highly specific direct protein–protein interactions. This would allow distinct ‘c-di-GMP control modules’ to operate in parallel and generate different outputs. Even ‘local c-di-GMP pools’ separate from the overall cellular c-di-GMP pool have been considered, but experimental evidence for this possibility has been lacking so far [2,3,26].

Highly specific direct contacts between DGCs, PDEs and their effector and target components have been amply documented [10,27–32]. Within these complexes, protein–protein interactions can have a *scaffolding* function, i.e. serve to establish close proximity between a DGC, i.e. a local source of c-di-GMP, and the thereby preferentially served c-di-GMP-binding effector component. A recently reported example is the DGC GcbB in *Pseudomonas fluorescens*, which directly docks onto the membrane-integral c-di-GMP effector LapD, which promotes biofilm formation by inhibiting the proteolytic release of a surface adhesin [29]. In addition, protein–protein interactions within a c-di-GMP signalling module can assume *regulatory* functions, i.e. directly activate or inhibit molecular functions of the partner proteins. This principle is illustrated by the PdeR/DgcM/MlrA complex in *E. coli* [30], which at the same time has provided the paradigm for a c-di-GMP-sensing trigger PDE (PdeR) and is therefore described in detail in §4.

## 3. c-di-GMP-binding effectors

**Figure 1.**
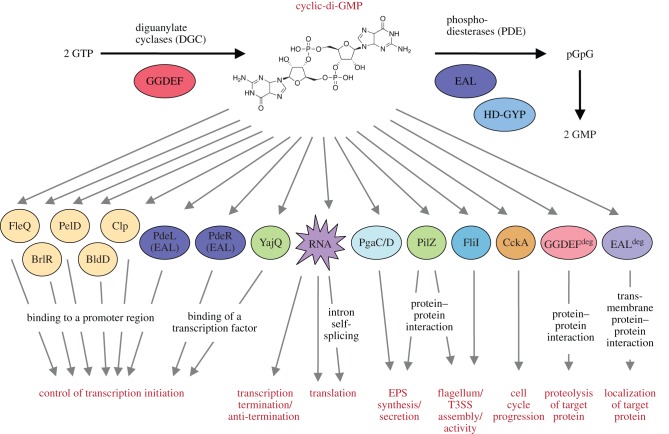
Diversity of c-di-GMP-binding effectors. Proteins (circles or ovals) that belong to a variety of different protein families as well as RNAs, i.e. 5′-untranslated regions of mRNAs (riboswitches; irregularly shaped star), can bind c-di-GMP with affinities ranging over three orders of magnitude (*K*_d_ between low nanomolar and low micromolar). Classical TFs (labelled in yellow) allosterically regulated by c-di-GMP include the AAA+ ATPase FleQ [33], PelD [34] and the MerR-like regulator BrlR [35] found in *Pseudomonas aeruginosa*, the CRP-like virulence regulator Clp in *Xanthomonas campestris* [36] and BldD, a master regulator of *Streptomyces* development [37]. YajQ [38] and PilZ [39–45] are small c-di-GMP-binding proteins or domains of larger proteins that serve as versatile adaptors or coupling factors between c-di-GMP and complex targets with diverse output functions. PgaC/D is a synthase and secretion system for the exopolysaccharide PGA in *E. coli* that consists of two membrane-integrated proteins whose interaction and therefore activity is stabilized by binding c-di-GMP [46]. FliI is a c-di-GMP-binding component of the *P. fluorescens* flagellum basal body serving as rotary export ATPase with similarly functioning homologues in other type III secretion systems [47]. While degenerate GGDEF and EAL domain proteins, which are enzymatically inactive but able to bind c-di-GMP, were recognized as c-di-GMP effectors quite a while ago [48,49], PdeR and PdeL are active PDEs now termed ‘trigger PDEs’, whose function as c-di-GMP sensing effectors has only been recently characterized [30,50] and is described in detail in §§5 and 6, respectively. Note that the array of c-di-GMP-responsive effector components is not exhaustive, but those mentioned here have been chosen to represent different families of proteins.

An intracellular second messenger such as c-di-GMP has to be sensed, i.e. bound by a specific effector component or receptor that interacts with a specific target to exert cellular effects. There is a striking diversity of c-di-GMP effectors and targets—as a consequence, virtually any kind of process in a bacterial cell can be controlled by c-di-GMP (figure 1). In principle, c-di-GMP, which can adopt different monomeric and dimeric conformations [23], can interact with RNAs, i.e. riboswitches, or diverse classes of proteins. Riboswitches are usually located in the untranslated 5′ regions of mRNAs (5′-UTR) and can fold into different conformations depending on c-di-GMP binding, which can promote or inhibit transcriptional termination, translation or even self-splicing of the mRNA [51–53].

Among the highly diverse c-di-GMP-binding effector proteins, PilZ proteins have been most thoroughly studied [23,40–42,54,55]. The PilZ domain, which can bind monomers or dimers of c-di-GMP, serves as a flexible adaptor domain that couples c-di-GMP-sensing to a variety of proteins that, for instance, serve to inhibit flagellar rotation or are part of exopolysaccharide synthesis and extrusion systems [43–45,56,57]. Also several types of bacterial transcription factors (TF) directly use c-di-GMP as a ligand for allosteric regulation of their activity and thereby control genes involved in biofilm matrix production, flagella expression, development or virulence [33,36,37,58]. The c-di-GMP-binding YajQ protein directly interacts with a LysR-like TF and seems to represent another type of adaptor that couples c-di-GMP sensing to an output activity, i.e. transcription initiation [38]. Additional recently identified c-di-GMP-binding proteins with highly specific functions are the exopolysaccharide synthase and secretion pore PgaC/PgaD in *E. coli* [46], the flagellum component and export ATPase FliI and the ribosomal modification protein RimK in plant-associated *Pseudomonas* species [47,59] and the proteinkinase/phosphatase CckA, which acts as a master regulator of cell cycle progression in *C. crescentus* [60].

Furthermore, degenerate GGDEF or EAL domains that can bind c-di-GMP, but are enzymatically inactive, can serve as c-di-GMP effectors. Examples include the degenerate GGDEF domain protein PopA in *C. crescentus*, which binds c-di-GMP via its intact I-site and thereby controls proteolysis of the cell cycle inhibitor and global developmental regulator CtrA [48], or the degenerate EAL domain protein LapD, the transmembrane biofilm regulator in *P. fluorescence* already mentioned above [61].

## 4. Trigger PDEs as a novel class of c-di-GMP-sensing effector proteins

Quite unexpectedly, a particular class of *enzymatically active* EAL domain proteins has recently been added to the growing list of c-di-GMP-sensing effectors, with PdeR (formerly YciR) of *E. coli* as the prototype [30]. These c-di-GMP-sensing ‘trigger PDEs’ are more than simple PDEs and in fact combine a number of functions: they (i) control the activity of a macromolecular target (another protein or a promoter region on the DNA) by direct and specific interactions in a manner that is modulated by (ii) their binding and degradation of c-di-GMP (i.e. their PDE function), which therefore represents (iii) the c-di-GMP sensor or effector function. Thus, their *primary* function is the control of activity of another macromolecule by direct interaction, while their PDE activity is a *secondary* function that modulates the primary activity. Appreciating the full function of these proteins thus requires a change of perspective, as initially we recognize and classify them as carriers of intact EAL domains and therefore just PDEs.

The multifunctionality of these trigger PDEs is reminiscent of bifunctional ‘moonlighting’ enzymes already observed many years ago (more recently summarized in [62]) and, in particular, a subclass of these, for which the name ‘trigger enzymes’ was proposed because they act as regulatory factors that trigger transcriptional responses [63]. These bifunctional enzymes (and in some cases transport systems) control gene expression via direct protein–protein or protein–DNA/RNA interactions in response to the availability of the substrates for their enzymatic (or transport) activities, with the substrates being central metabolites as illustrated by the following examples: (i) the proline-degrading enzyme PutA directly binds to and controls the activity of promoter regions of target genes [64]; (ii) the apo-form of the iron–sulfur cluster enzyme aconitase binds to iron-responsive elements in mRNAs encoding other tricarboxylic acid cycle enzymes [65]; (iii) the esterase Aes and the βC-S lyase MalY control the transcription factor MalT by direct interaction [66–68]; and (iv) the phosphotransferase system involved in glucose uptake also binds and sequesters the transcription factor Mlc [69]. In all cases, the enzymatic activities of these trigger enzymes modulate their direct interactions with other macromolecules (TF, DNA promoter regions or RNA) which results in a control of gene expression.

The c-di-GMP-specific PDE PdeR in *E. coli* seems the first trigger enzyme found to be involved in second messenger signalling, which makes it a ‘trigger PDE’ that acts as a novel type of c-di-GMP-sensing effector. In principle, its mode of operation is not so different from other effectors, which are allosterically controlled by c-di-GMP binding. The difference is the fact that a trigger PDE not only binds the ligand, but also degrades it, i.e. it possesses a mechanism to get rid of its ligand and even decrease its cellular concentration. Thus, a trigger PDE not only responds to c-di-GMP, but feeds back onto the level of c-di-GMP, which corresponds to an inherent negative feedback loop. This circuitry renders signalling through a trigger PDE more dynamic—a sustained response depends on continuous synthesis of c-di-GMP by at least one active DGC, since otherwise the trigger PDE would reduce the concentration of the signalling ligand (with kinetics depending on the actual cellular concentration and specific activity of the trigger PDE). On the other hand, a trigger PDE can accelerate switching off even multiple downstream responses to c-di-GMP (mediated by itself as well as by additional classical allosterically controlled effectors) when second messenger synthesis is reduced owing to changes in environmental or cellular conditions. If its direct control affects the activity of a key transcription factor, a trigger PDE is ideally suited to be *the* major switch component of a physiologically central signal transduction pathway or network.

With their direct and highly specific macromolecular interactions, trigger PDEs also represent a specific type of local c-di-GMP signalling in which a particular c-di-GMP-related enzyme generates a distinct individual output—without the need to postulate a ‘local c-di-GMP pool’ (see above). In this perspective paper, however, the focus is on their novel function as c-di-GMP-sensing effectors, which is described in detail in §§5 and 6 using the currently known two trigger PDEs that were both found in *E. coli*: (i) PdeR, which controls the activity of a *protein* target—a transcription factor—and whose analysis led to the trigger PDE concept as outlined above [30], and, more recently, (ii) PdeL, which binds to *DNA* and seems to control gene expression directly [50].

## 5. PdeR: a trigger PDE throws the switch to turn on biofilm matrix production

PdeR (formerly YciR) is the key player in the molecular switch that turns on biofilm matrix production in *E. coli*. The direct target of this c-di-GMP-mediated switch mechanism is the expression of the transcription factor CsgD, which is induced in planktonic culture as well as in biofilms when cells enter into stationary phase [6,7,30]. CsgD directly activates the transcription of *csgBAC*, i.e. the structural operon for amyloid curli fibre formation, and indirectly controls cellulose synthase activity by activating the expression of DgcC (formerly YaiC, or AdrA in *Salmonella*) [70].

The backbone of the regulation of CsgD expression is a feedforward transcription factor cascade that uses the stationary phase sigma factor RpoS (*σ*^S^) as a master regulator and the MerR-like transcription factor MlrA as a highly specific activator of transcription initiation at the *csgD* promoter [6,7,30]. MlrA activity is supported by DgcM (YdaM), which—besides producing c-di-GMP—also acts as a direct transcriptional co-activator [30]. c-di-GMP has a regulatory impact at two positions of this hierarchically organized biofilm control network [3]: (i) it controls the activity of MlrA and thereby CsgD expression, which affects the production of both curli fibres and cellulose, and (ii) further downstream in the network, it activates cellulose synthase specifically via DgcC (this additional cellulose-specific control allows cells to vary their curli : cellulose production ratio, i.e. the local composition of the matrix within the biofilm).

**Figure 2.**
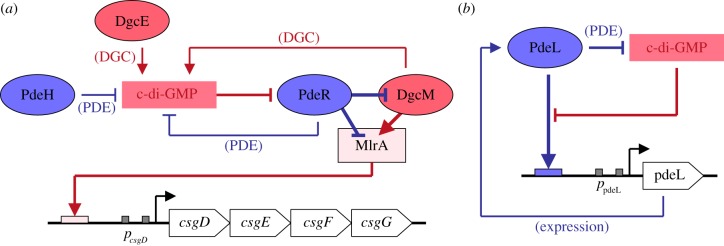
Regulatory circuits of gene expression exerted by the trigger PDEs PdeR and PdeL of *E. coli*. (*a*) At low cellular c-di-GMP levels, PdeR inhibits DgcM and the transcription factor MlrA by direct interaction and, as a consequence, the biofilm regulator CsgD is not expressed. The fact that PdeR also inactivates the inhibitor—i.e. c-di-GMP—of its own inhibitory action on DgcM/MlrA, sets up a positive feedback loop that stabilizes the inhibition of DgcM/MlrA by PdeR (i.e. the CsgD^OFF^ state). When c-di-GMP levels increase (e.g. during entry into stationary phase when the RpoS-dependent DgcE is induced, whereas PdeH is no longer expressed and its cellular level decreases), binding and cleavage of c-di-GMP by PdeR releases DgcM and MlrA. This allows DgcM to act as a direct co-activator for MlrA in the transcriptional activation of the *csgDEFG* operon and to also produce c-di-GMP (representing a positive feedback loop that stabilizes the CsgD^ON^ state) [30]. As a transcription factor, CsgD directly activates the expression of the subunits of amyloid curli fibres and indirectly stimulates the production of the exopolysaccharide cellulose. CsgE, CsgF and CsgG are components of the curli secretion machinery [71]. (*b*) PdeL activates its own expression in a manner that is inhibited by high c-di-GMP levels. In this circuit, two nested positive feedback loops seem to accelerate a decrease of the cellular c-di-GMP levels below a certain threshold: (i) positive autoregulation of PdeL and (ii) as a PDE, PdeL inactivates its own inhibitor c-di-GMP. This circuit could allow rapid and highly efficient switching to low cellular c-di-GMP levels and therefore a rapid stop of expression or activity of biofilm-related functions [50]. For additional details, see §§5 and 6.

The trigger PDE PdeR is the central component of the c-di-GMP switch that controls MlrA activity and thus CsgD expression. In response to the rising c-di-GMP level generated during transition into stationary phase by the RpoS-driven induction of the DGC DgcE (YegE) [7], PdeR allows the equally RpoS-dependent DgcM and MlrA to jointly activate *csgD* transcription by an intriguing mechanism (figure 2): it initially inhibits both DgcM and MlrA by direct specific interactions, which are relieved when c-di-GMP levels get high enough for PdeR to efficiently bind and degrade c-di-GMP. Thus, PdeR combines three activities: it is (i) a direct antagonist for DgcM and MlrA, (ii) a PDE and (iii) a sensor of the rising cellular c-di-GMP level during entry into stationary phase [30]. A key experimental hallmark in delineating this trigger mechanism was the possibility to separate these activities of PdeR genetically: a point mutation in the EAL motif (generated in the natural chromosomal copy of *pdeR* in order not to disturb stoichiometries of interacting partner proteins) eliminates PDE activity but does not affect its direct interactions—it thus converts PdeR into a *constitutive*, i.e. no longer c-di-GMP-responsive, ‘super-inhibitor’ of DgcM and MlrA, which completely eliminates CsgD and curli expression. By contrast, in a *pdeR* null mutant, expression of CsgD and curli production are very high, yet are equally ‘blind’ to any variation in the cellular c-di-GMP level [30].

Mathematical modelling indicated that the DgcE/PdeR/DgcM/MlrA-mediated c-di-GMP control module can operate as a bistable switch [72]. The major ingredients generating such behaviour are two feedback loops that stabilize the OFF and ON states, respectively, in *csgD* transcription (figure 2): (i) PdeR degrades the inhibitor, i.e. c-di-GMP, of its own inhibition of DgcM and MlrA and (ii) once DgcM gets released from this direct inhibition by PdeR (by sufficient c-di-GMP initially produced by DgcE), it contributes to accumulate c-di-GMP and thus further prevents PdeR from taking over again, i.e. from resuming its inhibition of DgcM and MlrA. This bistable switch is likely to be involved in generating the pronounced heterogeneity of matrix production in slowly growing zones of *E. coli* macrocolony biofilms [8,9,73].

Overall, the trigger PDE PdeR is the crucial factor for the decision whether—and where in a biofilm—cells produce extracellular matrix, which in turn generates the elaborate supracellular matrix architecture and thereby sometimes rather spectacular morphology of macrocolony biofilms.

## 6. PdeL: a trigger PDE directly controls gene expression

The *E. coli* protein PdeL (formerly YahA), which was one of the first EAL domain proteins shown to be an active c-di-GMP-specific PDE [74], carries an N-terminal LuxR-like domain with a helix–turn–helix (HTH) motif linked to its EAL domain. It is most strongly expressed in growing cells at 37°C, suggesting it may be relevant for *E. coli* within the human host [75]. Binding of c-di-GMP stimulates dimerization of the purified PdeL-EAL domain with the dimer interface promoting the formation of an active catalytic centre [76].

The presence of the potentially DNA-binding LuxR domain in combination with the c-di-GMP-binding and degrading EAL domain suggested PdeL could be a gene expression-controlling trigger PDE. This possibility raised a number of obvious questions. What is the target gene(s) under control of PdeL? And with a target gene identified, is there an influence of variations in the cellular c-di-GMP level on its regulation of target gene expression that depends on a functionally intact EAL domain? Or, in practical terms, would a point mutation, which eliminates PDE activity but leaves structure and interactions of PdeL intact, result in c-di-GMP-insensitive expression of the target gene?

In a recent report [50], PdeL was found to activate its own expression. It binds directly to an imperfect palindromic region relatively far upstream in the *pdeL* promoter region, which further downstream also features a binding site for the transcription factor Cra as noticed earlier [77]. Intriguingly, positive autoregulation by PdeL was observed only under conditions of low cellular c-di-GMP levels, i.e. was most pronounced in a strain with four DGCs knocked out, but still significant in a wild-type background, when cells were assayed *before* entry into stationary phase, where c-di-GMP levels increase [50,78]. These findings indicate that c-di-GMP binding and degradation somehow interfere with transcriptional activation by PdeL. This in turn may suggest that PdeL is able to oligomerize in two different configurations, i.e. a transcriptionally inactive dimer that is promoted by c-di-GMP-binding and an alternative dimer/oligomer that binds to DNA and promotes transcription as would be expected for a transcriptional regulator of the HTH family. This will have to be clarified in future structural investigations.

In regulatory terms, the negative effect of c-di-GMP on the expression of the c-di-GMP-degrading PdeL and the positive autoregulation of PdeL represents a combination of two positive feedback loops. This complex motif is likely to generate a steep OFF switch—when the c-di-GMP level decreases, PdeL can kick in and further accelerate the disappearance of c-di-GMP. Whether and when this is of physiological relevance has to be shown in further studies. Another interesting question is whether PdeL may regulate additional genes besides its own.

## 7. Conclusion and perspectives

A conceptual hallmark of the trigger PDE mechanism is the unexpected finding that certain *enzymatically active* EAL proteins can serve as a novel type of c-di-GMP-sensing effector protein. As a consequence, a potential trigger PDE and therefore effector function has to be considered for any PDE that is found to directly and specifically interact with some macromolecule, which can be a protein, DNA or RNA. In other words, the primary activity of such a PDE may be to control the function of the bound macromolecule in response to sensing—by binding and degrading—c-di-GMP.

There is no reason to believe that trigger PDEs should be restricted to signal transduction by c-di-GMP. Rather, they might occur also for other second messengers, in particular in species with multiple enzymes that produce and degrade a particular second messenger, as, for instance, observed for cAMP in alpha-proteobacteria or certain mycobacteria [79,80]. Such multiplicity seems to pave the way for the evolution of local signalling. First, simple scaffolding interactions with partner macromolecules can emerge, which may further evolve to have direct regulatory impact, which in turn may become the primary activity of a trigger PDE. The currently known trigger PDEs feature EAL domains, but HD-GYP domain proteins could possibly play a similar role. Another interesting question is whether also DGCs can act as trigger enzymes. Once activated, DGCs seem to operate under conditions of substrate saturation, since cellular levels of GTP are in the low millimolar range [81] and thus orders of magnitude higher than the usual *K*_m_ of DGCs (low micromolar). Thus, DGCs are unlikely to serve as GTP sensors, but it is conceivable that in some cases a control of the enzymatic reaction via the sensory input domain or c-di-GMP binding at the I-site of the GGDEF domain may also modulate a direct regulatory interaction with some target protein. In any case, it seems likely that the enzymes that make and break nucleotide second messengers will have more surprises in store for us.
